# Electroencephalographic correlates of Chronic Fatigue Syndrome

**DOI:** 10.1186/1744-9081-5-43

**Published:** 2009-10-06

**Authors:** Michael J Decker, Humyra Tabassum, Jin-Mann S Lin, William C Reeves

**Affiliations:** 1 Chronic Viral Diseases Branch, National Center for Zoonotic, Vector-borne Enteric Diseases, Centers for Disease Control and Prevention, 1600 Clifton Road, Mail Stop A-15, Atlanta, Georgia, USA

## Abstract

**Background:**

Unremitting fatigue and unrefreshing sleep, hallmark traits of Chronic Fatigue Syndrome (CFS), are also pathognomonic of sleep disorders. Yet, no reproducible perturbations of sleep architecture, multiple sleep latency times or Epworth Sleepiness Scores are found to be associated consistently with CFS. This led us to hypothesize that sleep homeostasis, rather than sleep architecture, may be perturbed in CFS. To probe this hypothesis, we measured and compared EEG frequencies associated with restorative sleep between persons with CFS and matched controls, both derived from a population-based sample.

**Methods:**

We evaluated overnight polysomnography (PSG) in 35 CFS and 40 control subjects. PSG records were manually scored and epochs containing artifact removed. Fast Fourier Transformation was utilized to deconstruct individual EEG signals into primary frequency bands of alpha, delta, theta, sigma, and beta frequency domains. The spectral power of each frequency domain for each sleep state was compared between persons with CFS and matched controls.

**Results:**

In persons with CFS, delta power was diminished during slow wave sleep, but elevated during both stage 1 and REM. Alpha power was reduced during stage 2, slow wave, and REM sleep. Those with CFS also had significantly lower theta, sigma, and beta spectral power during stage 2, Slow Wave Sleep, and REM.

**Discussion:**

Employing quantitative EEG analysis we demonstrate reduced spectral power of cortical delta activity during SWS. We also establish reduced spectral power of cortical alpha activity, with the greatest reduction occurring during REM sleep. Reductions in theta, beta, and sigma spectral power were also apparent.

**Conclusion:**

Unremitting fatigue and unrefreshing sleep, the waking manifestations of CFS, may be the consequence of impaired sleep homeostasis rather than a primary sleep disorder.

## Background

Chronic fatigue syndrome presents clinically as incapacitating physical and mental fatigue, frequently accompanied by unrefreshing sleep, impaired memory and concentration, and diffuse musculoskeletal pain [1]. An absence of characteristic clinical signs or diagnostic laboratory abnormalities further create a diagnostic challenge [1]. Adding to this complexity, the clinical picture of CFS is similar to that observed with sleep disorders [2].

Symptoms of CFS have been attributed to sleep disorders such as alpha-delta sleep [3], upper airway resistance syndrome [4], obstructive sleep apnea syndrome [5] and insomnia [6]. However, sleep disorders within CFS may also represent non-specific findings attributable to comorbid disease or ascertainment bias [7]. To assess the relationship between sleep disorders with symptoms of CFS, and avoid potential confounds associated with studying clinic-based patients, we recruited persons with CFS who were identified during the Centers for Disease Control and Prevention's CFS population surveillance of Wichita, Kansas [8]. During a 2-day in-hospital research study we evaluated nocturnal sleep characteristics with polysomnography (PSG) and daytime sleepiness with multiple sleep latency testing (MSLT) and questionnaires.

Our polysomnographic assessment of nocturnal sleep architecture, MSLT, and Epworth Sleepiness scores demonstrated no difference in sleep architecture or propensity for daytime sleepiness between community-based individuals with CFS and their matched controls [9,10]. This absence of overt sleep pathology and daytime sleepiness led us to concur with Mahold [7] and others [11] who suspect that unremitting fatigue and unrefreshing sleep, hallmark traits of CFS, do not reflect a primary sleep disorder. Rather, these symptoms may reflect perturbed sleep homeostasis [12]. To assess the biological plausibility of this hypothesis, we assessed EEG frequencies associated with restorative sleep in persons with CFS and compared them with their matched non-fatigued controls.

## Methods

This study received Institutional Review Board approval from the Centers for Disease Control and Prevention (CDC) and collaborating institutions. Administration of protocols adhered to U.S. Department of Health and Human Services human experimentation guidelines. All participants were over 21 years of age and provided written informed consent.

### Study population

The present study, conducted between January and July of 2003, evaluated participants with CFS and healthy controls followed from 1997 through 2000 in the Wichita CFS Surveillance Study [8]. The Wichita CFS Surveillance Study employed a random digit-dialing telephone survey to screen 56,146 adult residents, 18 to 69 years of age, living in Wichita in 1997. That survey identified 5,295 persons with fatigue persisting one month's duration or longer. Those individuals were asked to participate in a detailed telephone interview; 3,528 agreed to participate, along with a subset of 3,634 randomly selected non-fatigued (NF) controls. The detailed interview was used to identify cases with fatigue of 6 months duration or longer, who did not feel better after rest, who did not report any fatigue-associated medical or psychiatric conditions, and who reported at least four of the eight CFS case-defining symptoms (CFS-like cases) [1]. CFS-like cases were invited to participate in a clinical examination to confirm CFS by 1994 criteria [1]. Two Randomly selected NF controls matched to CFS-like cases based on age, sex, race, and body mass index were also asked to participate in the clinical evaluation. We followed this telephone interview cohort in 1998, 1999, and 2000 with telephone interviews and clinical evaluations.

### In-hospital study participants

In 2002, all subjects ever having met criteria for clinically confirmed CFS during the surveillance study were invited to participate in the current study. Thus, we invited the 70 people, who were classified as having CFS at least once during the 4-year surveillance study, to participate in the current study and 58 (83%) agreed. We randomly selected an equal number of surveillance participants who had unexplained fatigue for at least six months or longer, at least once during the 4-year surveillance study, but who did not meet full CFS criteria, and 59 (84%) were enrolled. Finally, we enrolled 60 non-fatigued control subjects who had participated in the same surveillance program, but did not exhibit any medical or psychiatric exclusion, and had never reported fatigue of at least 1-month duration. They were matched to CFS cases based on sex, age, race, and body mass index.

### Clinical evaluation

All subjects were admitted to a Wichita hospital research unit for 2 days. On admission, subjects underwent reevaluation in terms of current CFS symptoms and exclusionary conditions. At the hospital, 43 current CFS cases were confirmed. These consisted of past CFS cases that still had CFS, as well as new onset CFS cases derived from chronically fatigued persons who previously did not meet CFS criteria. Persons with insufficient symptoms of fatigue to be considered CFS, at the time of the in hospital study, were not included in this report. Because NF controls were individually matched to cases with a CFS diagnosis during the prior surveillance study, and this in-hospital study occurred two years later, and subjects were classified according to current diagnostic status, individual matching could not be maintained. However, the groups were demographically comparable. The mean age of the sample was 50.5 years and mean body mass index was 29.2.

### Classification

Participants were admitted to a Wichita hospital research unit for 2 days where they underwent a standardized review of past medical history, a standardized physical examination, and provided blood and urine for routine analysis [1,8]. To identify psychiatric conditions exclusionary for CFS, specifically trained and licensed psychiatric interviewers administered the Diagnostic Interview Schedule for current Axis I disorders. Subjects with no exclusionary conditions were considered to be CFS if they met criteria of the 1994 CFS case definition [1], as applied following recommendations of the International Chronic Fatigue Syndrome Study Group [8]. Subjects completed a series of rating scales to assess functional impairment (SF-36), fatigue severity (MFI), and occurrence, frequency and severity of the 8 CFS defining symptoms [13]. Classification as a current CFS case was based on cutoff scores in these rating scales with respect to the 3 dimensions of CFS specified in the case definition, i.e., impairment, fatigue, and accompanying symptoms [13]. Subjects meeting these criteria at the time of the study were classified as having CFS (n = 35) and those who met no criteria were classified as well (n = 40).

### Polysomnographic and Multiple Sleep Latency Techniques

Detailed descriptions of data collection techniques and protocols for nocturnal PSG and daytime multiple sleep latency testing (MSLT) have been described elsewhere [9,10]. Briefly, all PSG procedures were conducted within a 4-bed laboratory established at Wesley Medical Center, Wichita, KS. Each subject had a standard nocturnal PSG performed on the first night in the Medical Center, followed by MSLT the next day and a second PSG performed on the second night. Subjects were asked to arrive three hours before their typical bedtime on Night 1 to allow adequate time for electrode application and standard biocalibrations. Lights out and lights on times were standardized at 22:00 and 07:00, respectively, for all subjects.

During the PSG, standard gold cup electrodes were employed for the recording of electroencephalography (EEG), electroencephalography (EOG), and electromyography (EMG) in the following montage: central EEGs (C3-A2//C4-A1), occipital EEGs (O1-A1//O2-A2) EEGs, left and right monopolar EOGs, surface mentalis EMGs, and a three lead electrocardiogram. These signals were collected at a sampling rate of 200 Hz. Respiration was measured with inductance plethysmography-like belts placed around the chest and abdomen. A nasal cannula, attached to a pressure transducer, was positioned in close approximation to the nares to provide indices of airflow. A pulse oximeter probe, to measure hemoglobin oxygen saturation (Sp02), was applied to either the right or left index finger. Leg movement activity was measured via surface EMG electrodes applied to both the right and left anterior tibialis muscles.

### Scoring of polysomnography data

All PSG data was scored by a single registered polysomnology technologist who was blinded to each subjects' enrollment classification. Each PSG recording was manually scored in 30 second epochs, with each epoch scored as either wake, Stages 1, 2, slow wave, or rapid eye movement (REM) sleep. Criteria for scoring sleep and respiratory variables were upon based definitions used by the Sleep Heart Health Study [14].

### Fast Fourier Transformation of EEG Data

Following manual scoring of each subject's night one and two PSG recordings, the night two PSG was prepared for spectral analyses. Each 30-second epoch was assessed for the presence of EEG artifact (random high frequency noise attributed to movement arousal, etc), which was marked as movement artifact and excluded from further analysis. Removing entire epochs insured that the final FFT data output would be appropriately synchronized with each scored, artifact free, PSG epoch.

Following identification of all epochs containing artifact, we utilized the FFT algorithm within Somnologica Science (Embla, Denver Co., USA) to determine the spectral analysis profile of the EEG signal obtained between electrodes placed at the C3-A2 region of the scalp. The FFT algorithm was adjusted to deconstruct the EEG signal into the primary frequency domains described in Table 1 (Table 1). Since EEG signals were recorded at 200 Hz, each thirty second epoch contained 6,000 EEG data points per channel.

**Table 1:** primary EEG frequencies

**Frequency name**	**Frequency range** **(Hertz)**
Delta	0.5 - 3.99

Theta	4.0 - 7.99

Alpha	8.0 - 11.99

Sigma	12.0 - 13.99

Beta	14.0 - 24.99

The FFT analysis window processed 256 samples of EEG data as a signal unit, with each "unit" representing 1.28 seconds of EEG data. As the FFT analysis window progressed along the EEG signal, the final 128 data points within the preceding analysis window were included with the next 128 EEG data points, to be analyzed. This analysis routine produced 23.44 discrete FFT values for each 30 second epoch of EEG data. These 23.44 FFT values were then averaged into one FFT value representing the entire 30 second epoch. This methodological approach enabled us to define both the "power" within the alpha, delta, theta, beta and sigma frequency bands (Table 1) as well as the relative contribution that the power within each frequency provided to the overall power of the EEG signal.

### Medication use

Clinic staff reviewed all current medications (prescription and over the counter) that study participants were taking. All participants continued their medication use throughout the study. CDC CFS research program staff classified these medications as those affecting sleep (i.e., inducing sleep, inhibiting sleep or with mixed effects) or not affecting sleep. Medications that we encountered during this study included analgesics (e.g., hydrocodone, Lortab, oxycodone, Propoxyphene), antidepressants (e.g., Celexa™, amitriptyline, imipramine, Lexapro™, Wellbutrin™, Effexor, Prozac™, Zoloft™, Paxil™, fluoxetine), antianxiety (Alprazolam), antihistamines (e.g., diphenhydramine, chlorpheneramine, benadryl, promethazine), decongestants (e.g., pseudoephedrine, guaifenesen), anticonvulsants (e.g., Topamax, Neurotin, clonazepam), anti-sleep phase disorder (melatonin), blood pressure controlling (e.g., Clonidine, Proamatine), antipsychotics (e.g., Seroquel, Zyprexa, Fluvoxamine), stimulants (e.g., methylphenidate, Provigil), peristaltic stimulants (Metoclopramide), and muscle relaxants (cyclobenzaprine). Medications affecting sleep were handled as a binary measure (i.e., they used or did not use one or more of those named above). Analyses took into account use of sleep affecting medications.

### Statistical analysis

Spectral power for each frequency domain was determined for each 30 second epoch of sleep as well as wakefulness prior to sleep onset. Independent samples t-tests were employed to assess values of alpha, delta, theta, beta and sigma between control subjects with CFS subjects. Group differences were considered significant at a two-tailed significance of 0.05.

## Results

Seventy five of the 177 study participants met criteria as either CFS (n = 35) or NF control (n = 40). Remaining participants were excluded on the basis of significant medical or psychiatric exclusionary criteria that emerged during clinical evaluation, or else symptoms were insufficient to be classified as CFS. Table 2 (Table 2) provides demographics comparing non-fatigued males with CFS males and non-fatigued females with CFS females. Most participants were white women and no important differences existed between values of age or body mass index.

**Table 2:** demographics

**Males**	**CFS** **(n = 5)**	**Control NF** **(n = 4)**	**Two-tailed significance**
**BMI**	28.2 (5.4)	29.5 (5.7)	P = 0.740

**Age**	47.0 (0.7)	48.0 (10.9)	P = 0.841

**Race**	White = 5	White = 4	

			

**Females**	**CFS** **(n = 30)**	**Control NF** **(n = 36)**	**Two-tailed significance**

**BMI**	28.8 (4.1)	29.4 (8.1)	P = 0.715

**Age**	50.9 (9.1)	50.8 (8.1)	P = 0.988

**Race**	White = 27 AmIndian = 1 Multiracial = 1 Black = 1	White = 36	

Data presented as the mean ± 1 standard deviation of the mean

Values of sleep architecture measured during overnight PSG did not differ between NF controls and those with CFS, and those findings have been published previously [10]. Measures of physiologic sleepiness, as determined by the MSLT, also did not differ between groups [9]. In addition, medication use was similar between NF controls and those with CFS. Subjects who reported using any of the aforementioned medications routinely demonstrated experienced increased REM latencies (P = 0.02) and/or an increased percentage of Stage 1 sleep (P = 0.01). However, REM latency and percentage of Stage 1 sleep did not differ between the NF control group and CFS group [10].

PSG records from the 35 people with CFS and 40 non-fatigued controls were analyzed with the FFT algorithm. Table 3 illustrates that spectral power of cortical alpha activity was significantly reduced in CFS subjects during Stage 2 Sleep, Slow Wave Sleep (SWS), and REM (Table 3). Table 4 denotes that spectral power of cortical delta activity was also significantly reduced in SWS but increased in both Stage 1 sleep and REM (Table 4). Similarly, Table 5 demonstrates that spectral power of cortical theta activity was significantly reduced in Stage 1, 2, and Slow Wave Sleep (Table 5). Finally, Table 6 illustrates that spectral power of cortical sigma activity was significantly reduced in Stage 2, SWS, and REM (Table 6) while spectral power of cortical beta activity was significantly reduced in Stage 2 Sleep, Slow Wave Sleep (SWS), and REM (Table 7).

**Table 3:** alpha power

	**Stage 1**	**Stage 2**	**Slow Wave Sleep**	**REM**
CFS (n = 35)	1.17E-09 ± 1.45E-11	1.42E-09 ± 6.59E-12	1.525-09 ± 1.44E-11	6.84E-10 ± 5.95E-12

Control NF (n = 40)	1.22E-09 ± 1.86E-11	1.73E-9 ± 1.01E-11	2.07E-09 ± 2.43E-11	8.76E-10 ± 9.50E-12

Two Tailed Significance	P = 0.058	P < 0.0001	P < 0.0001	P < 0.0001

Data presented as the mean ± 1 standard error of the mean

**Table 4:** delta power

	**Stage 1**	**Stage 2**	**Slow Wave Sleep**	**REM**
CFS (n = 35)	4.87E-9 ± 7.16E-11	1.17E-08 ± 5.26E-11	3.48E-08 ± 2.29E-10	3.76E-09 ± 2.73E-11

Control NF (n = 40)	4.22E-9 ± 5.86E-11	1.16E-08 ± 8.02E-11	3.83E-08 ± 2.36E-10	3.54E-09 ± 5.35E-11

Two Tailed Significance	P < 0.0001	P = 0.323	P < 0.0001	P < 0.0001

Data presented as the mean ± 1 standard error of the mean

**Table 5:** theta power

	**Stage 1**	**Stage 2**	**Slow Wave Sleep**	**REM**
CFS (n = 35)	1.57E-9 ± 1.65E-11	2.35E-09 ± 8.98E-12	3.52E-09 ± 1.89E-11	1.36E-09 ± 8.53E-12

Control NF (n = 40)	1.40E-09 ± 1.26E-11	2.41E-09 ± 7.78E-12	3.75E-09 ± 1.61E-11	1.37E-09 ± 8.71E-12

Two Tailed Significance	P < 0.0001	P < 0.0001	P < 0.0001	P = 0.228

Data presented as the mean ± 1 standard error of the mean

**Table 6:** sigma power

	**Stage 1**	**Stage 2**	**Slow Wave Sleep**	**REM**
CFS (n = 35)	2.98E-10 ± 3.77E-12	4.86E-10 ± 2.41E-12	3.65E-10 ± 2.51E-12	1.51E-10 ± 1.38E-12

Control NF (n = 40)	2.91E-10 ± 3.59E-12	5.42E-10 ± 2.56E-12	4.22E-10 ± 3.10E-12	1.82E-10 ± 1.61E-12

Two Tailed Significance	P = 0.180	P < 0.0001	P < 0.0001	P < 0.0001

Data presented as the mean ± 1 standard error of the mean

**Table 7:** beta power

	**S1**	**S2**	**SWS**	**REM**
CFS (n = 35)	8.35E-10 ± 1.13E-11	7.51E-10 ± 3.65E-12	4.77E-10 ± 3.69E-12	4.75E-10 ± 4.01E-12

Control NF (n = 40)	8.46E-10 ± 9.81E-12	8.44E-10 ± 3.41E-12	4.90E-10 ± 3.02E-12	5.37E-10 ± 4.29E-12

Two Tailed Significance	P = 0.459	P < 0.0001	P = 0.004	P < 0.0001

Data presented as the mean ± 1 standard error of the mean

## Discussion

We sought to determine whether the power spectra of EEG frequencies associated with restorative sleep differed between persons with CFS and matched controls. Employing quantitative EEG analysis we demonstrate reduced spectral power of cortical delta activity during SWS. We also establish reduced spectral power of cortical alpha activity, with the greatest reduction occurring during REM sleep. Reductions in theta, beta, and sigma spectral power were also apparent. In contrast, the percentage of PSG recording time spent awake, in light sleep, slow wave and REM sleep, as well as daytime propensity for sleepiness, did not differ between persons with CFS and their matched controls [9,10].

Alpha activity is the primary EEG frequency associated with wakefulness and vigilance [15-17]; alpha activity increases following administration of wake promoting drugs [18] and is attenuated during cortical quiescence [19,20]. Delta activity is the primary EEG frequency associated with slow wave sleep (21). Increased delta spectral power occurs immediately following sleep restriction or deprivation, and is postulated to reflect a homeostatic sleep rebound. Attenuation of delta power is associated with impaired sleep homeostasis [21].

Experimentally reduced delta spectral is followed by increased perception of pain, generalized discomfort and fatigue [22,23]. Key executive functions such as memory consolidation also are negatively impacted [24,25]. Metabolic dysfunctions emerge as decreased insulin sensitivity, inadequate increase in subsequent insulin release, and reduced glucose tolerance [26]. Sympathovagal imbalance arises and sympathetic tone dominates, as determined by diminished heart rate variability [26]. These sensory, cognitive, metabolic and sympathovagal dysfunctions are alleviated when delta spectral power is permitted to return to baseline levels [26].

We speculate that reduced spectral power of cortical delta and alpha activity observed in CFS signify impaired sleep homeostasis. The co-occurrence of fatigue, generalized pain, discomfort, impaired memory, and insulin resistance observed between persons with CFS and healthy control subjects with experimentally attenuated delta power is striking. Yet, the 10% reduction of delta spectral power within those with CFS may be considered modest in comparison with the 50-70% reductions employed to elicit sensory and physiologic responses within healthy subjects [26]. This compels us to postulate that the cumulative effects of chronic, but modest, attenuations of delta spectral power evokes outcomes similar to those appearing after a brief, but significant suppression.

Data presented here converge with that from another recent study demonstrating reduced spectral power of delta EEG activity within CFS subjects following sleep restriction [12]. Yet, results from our convergent studies diverge from that of an earlier study demonstrating increased alpha activity in CFS, particularly during SWS [2]. In that study, CFS subjects were recruited from a clinic-based program, which raised their likelihood of co-morbid medical conditions and subsequent biasing of results. The small sample size and qualitative approach for measuring and quantifying alpha activity employed in that study may also have contributed to these paradoxical findings.

Our observations corroborate those of Mahold [7] who proposed that unremitting fatigue and unrefreshing sleep, hallmark traits of CFS, do not reflect the presence of a primary sleep disorder. Our findings also confirm and extend recent observations of Armitage [12] who proposed that impaired sleep homeostasis contributes to the signs and symptoms of CFS. Nonetheless, a plausible alternate hypothesis is that sympathovagal imbalance may account for our findings as well as key signs and symptoms of CFS [27-29].

Sympathovagal imbalance with sympathetic predominance, expressed as reduced heart rate variability, existed in those CFS subjects studied here [30]. Increased sympathetic tone is associated with reduced spectral power of alpha activity during wakefulness [31,32]. However, it remains uncertain whether increased sympathetic tone also suppresses delta spectral power during sleep. Thus, attenuated delta power resulting from impaired sleep homeostasis, rather than a primary disorder of sympathovagal imbalance, remains the more parsimonious hypothesis to guide future studies eliciting mechanisms contributing to our findings.

## Limitations

Interactions between both psychotropic and non psychotropic medications with central nervous system activity are complex and extensive. Thus, it is feasible to consider that medication use may have potentially contributed to our findings. However, the risk for adverse effects associated with abrupt withdrawal of prescribed medications contributed to our a-prior decision to avoid restricting medication use prior to PSG. Future studies will be necessary to determine whether medications routinely prescribed to counter symptoms of CFS are associated with attenuated alpha and delta spectral power.

Perturbations of EEG spectral power also correspond with neurodegenerative disease [33], acute depression [34], emotional stress [35], diabetes [36], and medication [37]. To, reduce the likelihood of this, all study participants underwent full clinical evaluations to insure the absence that psychiatric or medical disorders, or medication effects, that could potentially confound our results. Thus, we are confident that the attenuation in the spectral power of delta and alpha activity observed in CFS is not attributed to psychiatric or medical pathology nor represent the effect of pharmaceuticals.

Other limitations also reflect aspects of study design. Our participants represented a population based sample, minimizing recruitment bias. However, those with CFS had been ill for more than 5-years, so survival bias must be considered. Participants on the whole were overweight or obese, so the results cannot be generalized to those of normal BMI. Obesity is also associated with stimulation of several important inflammatory pathways. This was controlled for by the matched case control design and statistical analysis, but future studies should be designed to evaluation inflammatory markers in some detail. Additionally, most participants were older women and this study did not include data on menopausal status, which has an important impact on sleep. Our matched case-control study design and statistical analyses largely corrected for effect of menopausal status on the results. However, as with the other limitations discussed above, further studies evaluating the effect of menopausal status must be conducted to explore this.

## Conclusion

We appreciate that data derived through our experimental paradigm cannot confirm the hypothesis that impaired sleep homeostasis and increased cortical quiescence exist within CFS. Future studies using methods known to augment delta and alpha spectral power, such as meditation [38], graded exercise [39] or administration of gamma hydroxybutyrate [40], followed by assessment of EEG spectral power and symptom expression are required to address this. Outcomes from those studies will not only provide new insight into potential mechanisms contributing to the signs and symptoms of CFS, but also potentially define new therapeutic interventions to restore sleep homeostasis in select patient populations.

## Competing interests

The authors declare that they have no competing interests.

## Authors' contributions

MJD directed polysomnographic data collection, and all data analyses and interpretation. HT performed FFT transformation and subsequent generation of the database. JSL validated statistical procedures and confirmed accuracy of statistical analyses. WCR directed the overall project and coordinated data collection. All authors have read and approved the final manuscript.
